# Outcomes and Trends of Open Thoracotomy and Video-Assisted Thoracic Surgery for Surgical Management of Catamenial Pneumothorax: A 10-Year Retrospective Cohort Study of a Thai Population

**DOI:** 10.7759/cureus.74083

**Published:** 2024-11-20

**Authors:** Pacharadanai Charoensup, Thansit Srisombut, Rada Thiannitiworakun, Padet Tanangterapong

**Affiliations:** 1 Department of Cardiovascular Thoracic Surgery, King Chulalongkorn Memorial Hospital, Bangkok, THA; 2 Department of Surgery, Sawanpracharak Hospital, Bangkok, THA; 3 Department of Surgery, King Chulalongkorn Memorial Hospital, Bangkok, THA

**Keywords:** bleb, catamenial pneumothorax, diaphragmatic lesion, endometriosis, pleurectomy

## Abstract

Background

Catamenial pneumothorax (CP) is characterized by pneumothorax associated with menstrual cycles and thoracic endometriosis. This study aimed to review the outcomes and trends for surgical treatment of CP in King Chulalongkorn Memorial Hospital.

Methodology

We included females aged 18 to 50 years who underwent surgery for CP between January 2012 and December 2022. A total of 17 patients were identified. A retrospective data collection from each patient was done, including demographic data, surgery type (open vs. video-assisted thoracic surgery (VATS)), pleural procedures, length of stay, and complications.

Results

The mean age at surgery was 36.29 years (±6.78). The median follow-up period was 36 months (range = 12-122). Pneumothorax occurred predominantly on the right side (94.12%), and pelvic endometriosis was present in all patients. All 17 patients underwent surgery, with open surgery in six and VATS in 11 patients. Diaphragmatic procedures were performed in 15 patients, with pleurectomy in nine, lung resection in 11, and pleurodesis in 10 patients. Pleurectomy was significantly associated with a reduced recurrence rate (p = 0.029). Diaphragmatic lesions were absent in two cases, and four patients experienced recurrence.

Conclusions

This cohort study of CP in the Thai population demonstrated that pleurectomy is associated with lower recurrence. There was no difference in recurrence between open surgery and VATS. Corresponding to the British Thoracic Society Guideline 2023, pleurectomy may help reduce recurrence.

## Introduction

Endometriosis most commonly involves the pelvis, particularly the ovaries, cul-de-sac, broad ligaments, and uterosacral ligaments. However, endometrial tissue can be found outside the pelvis in the abdomen, thorax, brain, and skin [1,2]. Thoracic involvement is the most frequent extrapelvic location of endometriosis and often presents as catamenial pneumothorax (CP) [3].

CP is characterized by spontaneous, recurrent pneumothorax associated with menstruation. It was first described by Maurer and colleagues in 1958 [4]. Primary spontaneous pneumothorax occurs in 5-10 per 100,000 patients. The general prevalence of CP in pneumothorax occurring in women of reproductive age ranges from 7.3% to 36.7% [5]. Historically, CP was associated with a high rate of recurrence, despite hormonal and surgical treatments [6]. Recurrence may be as high as 8% to 40% in patients undergoing surgery [7]. However, in recent years, the implementation of pleurectomy in the management of CP has changed the standard of treatment, leading to lower recurrences [8].

In the past decade, there has been a notable shift in the surgical approach to treating CP, with a growing preference for video-assisted thoracic surgery (VATS) due to less invasiveness and improved visualization. Surgical procedures involve the removal of lung blebs, endometrial nodules, and pleural interventions (pleurodesis, pleurectomy). Furthermore, a critical aspect of the surgical procedure involves the thorough examination and addressing of diaphragmatic pathology in CP [9]. Several factors that might be related to recurrence after surgery in patients with CP include a larger diaphragmatic defect, multiple sites of intrathoracic endometriotic spots, and the absence of postoperative hormonal therapy [10,11]. However, there are currently limited studies (case series) examining the prognostic factors and disease characteristics in the Thai population [12].

In this study, we performed a retrospective analysis of 17 patients with CP to determine the clinical characteristics, treatment outcomes, and recurrences. Additionally, we aimed to evaluate the factors associated with postoperative recurrences.

## Materials and methods

Study design and setting

**Figure 1:**
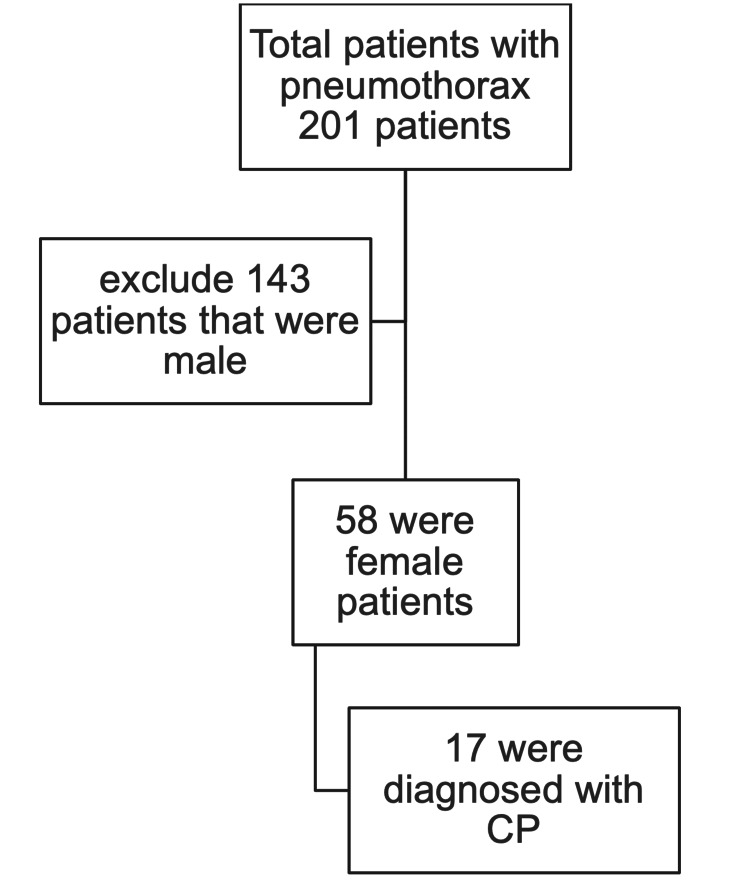
Number of patients who underwent surgery for pneumothorax at the King Chulalongkorn Memorial Hospital from January 2012 to December 2022. CP: catamenial pneumothorax

Ethical approval was granted by the Institutional Review Board of King Chulalongkorn Memorial Hospital (KCMH) (approval number: 0091/67). The study was a single-center, retrospective cohort study conducted at KCMH. We reviewed clinical and pathological data from operative records in the Department of Cardiovascular Thoracic Surgery between January 2012 and December 2022. A total of 201 patients with pneumothorax underwent surgery from January 2012 until December 2022, of whom 58 patients were female. A total of 17 patients were diagnosed with CP according to operative records (Figure 1). Diagnosis was done by clinical chest pain, dyspnea, and hemoptysis within 72 hours before and 72 hours after the onset of menses [13]. Clinical, pathological, and treatment data were collected to analyze treatment outcomes and recurrence.

Selection of participants

Participants were identified through an electronic database. All CP patients who underwent surgical treatment with records from operative notes (postoperative diagnosis) at our institution were included in the study cohort. The inclusion criteria were patients diagnosed with CP, aged between 18 and 50 years, who had received surgical intervention. The exclusion criteria included patients with chronic lung disease, a history of previous thoracic surgery, and those with a follow-up period of less than 12 months. The follow-up period was recorded from the day of surgery to the last follow-up visit. Routine follow-ups were scheduled at three months, six months, 12 months, and then annually.

Definition of catamenial pneumothorax and surgical procedure

CP is defined as pneumothorax related to menstruation, occurring within 72 hours before or 72 hours after the onset of menses, and is pathologically confirmed as thoracic endometriosis [13]. At our institution, the choice of surgical approach, whether thoracotomy or VATS, was determined by the surgeon. All procedures were conducted under general anesthesia with single-lung ventilation, and preoperative antibiotic administration was a standard protocol for all patients. Before surgery, patients were positioned in the lateral decubitus position.

During surgery, a meticulous examination of the lung was performed to identify and manage blebs, bullae, and potential endometriosis on the lung, pleura, and diaphragm, including diaphragmatic fenestrations. Any additional findings were addressed, including diaphragmatic repair, lung resection, pleurodesis, pleurectomy, and the excision of endometriotic lesions. Samples of thoracic endometriosis and blebs were sent for pathological confirmation. Postoperatively, all patients were referred to a gynecologist for further evaluation of endometriosis-related issues and consideration of postoperative hormonal therapy.

Data collection

We collected the following baseline characteristics: age, side of pneumothorax, history of pre- and postoperative hormonal treatment, smoking status, presenting symptoms, previous pregnancies, type of endometriosis, and follow-up time. Moreover, treatment variables including surgical approaches, pleural procedures, intraoperative findings, and pathological information were recorded. Outcomes included recurrence, postoperative variables such as duration of chest tube placement, length of hospital stay, and treatment details for recurrent cases.

Statistical analysis

Baseline characteristics of all patients were recorded and analyzed. The normality of the data was assessed using the Kolmogorov-Smirnov and Shapiro-Wilk tests. Continuous variables were presented as mean ± standard deviation (SD), median, and range as appropriate. Categorical data were presented as frequency and percentage. Comparisons between categorical variables were performed with chi-square tests or Fisher’s exact probability tests as appropriate. Continuous data were compared using Student’s t-test or Mann-Whitney U test as appropriate. A p-value <0.05 was considered to indicate statistical significance. All analyses were performed using STATA version 18 (StataCorp LLC College Station, TX, USA).

## Results

In this cohort of CP patients (n = 17), we examined intraoperative findings and treatments (Tables 1, 2) comparing individuals with recurrence (n = 4) to those without recurrence (n = 13).

**Table 1:** Baseline characteristics of catamenial pneumothorax patients. ^†^: the p-value was obtained from Fisher's exact test. ^‡^: the p-value was obtained from the Mann–Whitney U test. GnRH: gonadotropin-releasing hormone; DIE: deeply infiltrating endometriosis

Clinical characteristic	Total (17)	Recurrence (4)	Non-recurrence (13)	P-value
Age, years (mean ± SD)	36.29 (6.78)	33 (9.2)	37.31 (5.95)	0.28
Gender, female, n (%)	17 (100)	4 (100)	13 (100)	1
Side				1^†^
Bilateral	0	0	0	
Right	16 (94.12)	4 (100)	12 (92.31)	
Left	1 (5.88)	0	1 (7.69)	
Previous hormonal treatment, n (%)				
None	9 (52.94)	3 (75)	6 (46.15)	0.576^†^
GnRH agonist	0	0	0	-
Estrogen–progesterone	8 (47.06)	1 (25)	7 (53.85)	0.576^†^
Smoking status, n (%)				
No	17 (100)	4 (100)	13 (100)	-
Active	0	0	0	-
Previous	0	0	0	-
Presentation symptoms, n (%)				0.128†
Chest pain	10 (58.82)	1 (25)	9 (69.23)	
Dyspnea	6 (35.29)	2 (50)	4 (30.77)	
Cough	1 (5.88)	1 (25)	0	
Hemothorax	0	0	0	
Median follow-up time, months (range)	36 (12–122)	41 (35–55)	31 (12–122)	0.365^‡^
Previous pregnancy, n (%)	4 (23.53)	1 (25)	3 (23.08)	1^†^
Type of endometriosis, n (%)				
Superficial	8 (47.06)	1 (25)	7 (53.85)	0.576^†^
Endometrioma	10 (58.83)	4 (100)	6 (46.15)	0.103^†^
DIE	0	0	0	-

**Table 2:** Intraoperative findings and treatments received. ^†^: the p-value was obtained from Fisher's exact test. ^‡^: the p-value was obtained from the Mann–Whitney U test. GnRH: gonadotropin-releasing hormone; VATS: video-assisted thoracic surgery

Variable, (%)	Total (17)	Recurrence (4)	Non-recurrence (13)	P-value
Intraoperative findings				
Bleb	4 (23.53)	2 (50)	2 (15.38)	0.219^†^
Diaphragmatic defect	15 (88.24)	3 (75)	12 (92.31)	0.426^†^
Combined bleb and diaphragmatic defect	3 (17.65)	2 (50)	1 (7.69)	0.121^†^
Adjuvant hormonal treatment				
None	2 (11.76)	0	2 (15.38)	1^†^
GnRH agonist	2 (11.76)	0	2 (15.38)	1^†^
Estrogen–progesterone	13 (76.47)	4 (100)	9 (69.23)	0.519^†^
Operative treatment				
Diaphragmatic Procedure	15 (88.24)	3 (75)	12 (92.31)	0.426^†^
Pleurectomy	9 (52.94)	0	9 (69.23)	0.029^†^
Lung Resection	11 (64.71)	2 (50)	9 (69.23)	0.584^†^
Pleurodesis	10 (58.82)	3 (75)	7 (53.85)	0.603^†^
Operative approach				0.584^†^
Open	6 (35.29)	2 (50)	4 (30.77)	
VATs	11 (64.71)	2 (50)	9 (69.23)	
Median chest tube duration, days (range)	9 (5–38)	11 (5–30)	9 (6–38)	0.608^‡^

Regarding baseline characteristics, the mean age of the participants was 36.3 years. CP mostly occurred on the right side, with only one patient having left-sided CP. Previous hormonal treatment with estrogen-progesterone was noted in eight patients while the rest had no treatment. None of the participants were active or previous smokers. Presenting symptoms included chest pain (58.8%), dyspnea (35.3%), and cough (5.88%). Overall, 23.5% of patients were previously pregnant. These patients underwent a gynecologic examination and were found to have endometriosis, which was categorized as either superficial in eight patients (47.1%), endometrioma in 10 patients (58.8%), and none had deep infiltrating endometriosis (DIE). DIE is endometriosis which infiltrates more than 5 mm into the subperitoneal area. Comparing the baseline characteristics between the recurrence and non-recurrence groups, there was no significant difference.

The overall median follow-up time was 36 months (range = 12-122 months). In the recurrence group, the median follow-up time was 41 months (range = 35-55 months) compared to 31 months (range = 12-122 months) in the non-recurrence group. The difference was not statistically significant (p = 0.356).

On the other hand, intraoperative findings consisted of blebs, diaphragmatic defects, and combined blebs and diaphragmatic defects. Blebs were present in 23.5% of all cases, with a higher incidence in the recurrence group (50%) compared to the non-recurrence group (15.38%). Overall, 88.24% of cases had diaphragmatic defects, with no significant difference between the recurrence group (75%) and the non-recurrence group (92.31%) (p = 0.426). The combined finding of blebs and diaphragmatic defects was noted in 17.65%, occurring in 50% of the recurrence group and 7.69% of the non-recurrence group. This difference was not statistically significant (p = 0.121).

Operative treatments included diaphragmatic procedures (88.24%), lung resection (64.7%), pleurodesis (58.8%), and pleurectomy (52.9%). Diaphragmatic procedures were performed in 75% of the recurrence group and 92.3% of the non-recurrence group, with no statistically significant difference (p = 0.426). The choice of operative approach (open thoracotomy vs. VATS) showed no statistically significant difference between the groups.

On the other hand, pleurectomy was found to be significantly associated with non-recurrence as all nine (52.9%) patients who underwent pleurectomy had no recurrence (p = 0.029).

For treatment outcomes, the median duration of chest tube insertion was nine days (range = 5-38) for all patients, with a longer duration in the recurrence group (median = 11 days, range = 5-30) compared to the non-recurrence group (median = 9 days, range = 6-38), although this difference was not statistically significant (p = 0.608).

Regarding length of hospital stay, the median duration was nine days (range = 5-38) (Table 3). Of the 17 patients, eight had hospital stays longer than nine days. Factors contributing to longer hospital stays included pleural effusion and prolonged air leaks. Among recurrence cases (n = 4), 25% were managed conservatively with non-invasive measures, 25% underwent chest tube drainage, and 50% required surgical intervention. Prolonged air leak was observed in 12.5% (n = 2) of these cases. Two patients had an extended hospital stay due to delays in the operating room schedule during the preoperative phase.

**Table 3:** Length of stay and recurrence treatment. Prolonged air leak is characterized by a duration of seven days or more. LOS: length of stay

Variable (%)	Total (17)
Median LOS, days (range)	9 (5–38)
Recurrence treatment (N = 4)	
Conservative	1 (25)
Aspiration	0
Chest tube drainage	1 (25)
Surgical	2 (50)
Prolonged air leak	2 (12.5)

## Discussion

In this retrospective cohort study of 17 patients diagnosed with CP, we aimed to identify the demographic characteristics and treatment trends within the Thai population. Our findings indicate a demographic profile and treatment trends similar to those reported in the existing literature [14,15]. Pleurectomy showed a significant association with non-recurrence (p = 0.029). From a physiological standpoint, pleurectomy increases adhesion and fibrosis which obliterate the pleural space and potential for recurrence and air leakage [8]. Therefore, higher power in the data from longitudinal and multicenter studies might increase efficacy.

Demographics and characteristics

The typical age of presentation for spontaneous pneumothorax in CP ranges from 34 to 37 years [16]. In our study, participant ages ranged from 27 to 49 years, with a mean age of 36.29 years. The right-sided predominance of CP in our study (94.12%) is consistent with previous studies, which reported a rate of about 96.3% [6]. This supports the theory of air passing transdiaphragmatically from the genital tract due to diaphragmatic perforations caused by endometrial implants [16,17]. The age range of patients is consistent with previous reports of endometriosis, with the disease affecting reproductive-aged females [18].

In our cohort, a comparison of previous pregnancies revealed no significant differences between the two groups. To date, data indicate no association between the impact of previous pregnancies and the severity or recurrence rate of CP [19,20]. However, insights from endometriosis-related trials suggest that pregnancy has often been considered to exert a positive influence, attributed to factors such as anovulation, amenorrhea, and hormonal changes [19]. It is crucial to note that this positive effect is usually temporary, as disease activity tends to return [21]. Consequently, the recurrence of CP may not be significantly influenced by previous pregnancies. Future large clinical data are still needed to support this finding.

Pathophysiology of catamenial pneumothorax

Concerning pathophysiology knowledge, CP may originate from migration through diaphragmatic fenestrations in individuals with pre-existing diaphragmatic defects [10]. Surgeons are more vigilant in finding diaphragmatic lesions when dealing with right-sided lesions [10]. These endometriotic tissues are scattered around the pleural cavity, with some being small and not visible. Therefore, treatment guidelines indicate that surgical pleurodesis should be performed. Pleurodesis consists of chemical pleurodesis, pleural abrasion, and pleurectomy [13]. Each method stimulates inflammation and fibrosis between the lung and chest wall. However, pleurectomy is a choice because the lesion can be removed [8]. Moreover, pleurectomy facilitates intense inflammation compared to other methods making the parietal pleural and visceral pleura adherent to one another [8].

It is important to note that diaphragmatic fenestrations and endometriotic deposits cannot be identified in all CP cases. In our series, diaphragmatic defect, despite 15 (88.24%) patients presenting with diaphragmatic defects, including diaphragmatic fenestrations and endometriosis deposited at the diaphragm. Bleb or bullae were found in 23.53% of cases.

Treatment and surgical management

Postoperative hormonal treatment was recommended to all patients, but only two did not receive it due to contraindications. In total, 12 patients were treated with estrogen-progestin therapy, while two received a gonadotropin-releasing hormone (GnRH) agonist for six months. GnRH agonists suppress ovarian hormones, leading to amenorrhea [2]. Low doses of GnRH agonists should be combined with low-dose progestins to reduce climacteric-like symptoms and improve tolerability and adherence to therapy [22]. Most experts recommend administering this therapy in the immediate postoperative period for 6-12 months in all patients with confirmed catamenial or endometriosis-related pneumothorax [2]. An extended duration of at least 18 months, including hormonal therapy and surgery, is considered to prevent recurrence [2]. Low-dose oral contraceptives (estrogen-progestin) can also be used to manage endometriosis, though these treatments do not eradicate the disease without surgical removal of endometriotic foci [23]. Furthermore, one-third of women with endometriosis may not respond to estrogen-progestins, possibly due to progesterone resistance [22,24].

In our practice, the preferred approach was minimally invasive and performed through a thoracoscopy. VATS provides magnification and exposure of possible defects that are sometimes better than that provided by thoracotomy. Missed spots may occur, especially if the patient is positioned for an axillary thoracotomy as complete visualization of the diaphragm is difficult. A better approach seems to be VATS with the patient positioned for a posterolateral thoracotomy. Our approach to CP is a diaphragmatic resection in combination with mesh insertion in some cases, wedge resection, resection of any suspicious nodule, and pleurectomy.

In our cohort, every patient with an intraoperative finding of an endometriotic spot on the pleura underwent a pleurectomy, which has been shown to reduce recurrence rates in patients with CP [14]. Furthermore, the integration of thoracoscopy has played a pivotal role in enhancing the inspection of the pleura for detecting endometriotic tissue. The likelihood of partial or subtotal removal of endometriotic tissue during pleurectomy is unlikely. The primary concern arises when challenging angles during the procedure pose difficulties in identifying lesions, potentially leading to missed spots and, consequently, recurrence of CP. The absence of a significant difference in recurrence between VATS and thoracotomy in our cohort is likely attributed to the use of thoracoscopy in all cases.

Limitations

Several limitations need to be addressed in this study. First, due to its retrospective nature, some data were not available such as intraoperative findings of lesion localization. Second, the low number of cases, attributable to the rarity of CP, led to lower power. A power analysis yielded a statistical power of only 70%. To our knowledge, this is the first study of CP in the Thai population to evaluate disease characteristics.

Future directions

Pleurectomy, significantly associated with non-recurrence, had been optional. Should pleurectomy be done in all cases? A longitudinal study involving patients aged 10-20 years should be conducted. Moreover, a multicenter study may be feasible to increase the number of participants despite the rarity of this disease. On the other hand, visualization gain from VATS may decrease the recurrence of CP. However, there is no data on robotic-assisted thoracoscopic surgery that may improve precision and effectiveness.

## Conclusions

We have successfully demonstrated a significant reduction in CP recurrence after pleurectomy. Factors that might be associated with recurrence are the use of hormones, the presence of endometrioma, the presence of blebs, or a combination of blebs and diaphragmatic defects. However, there were no differences in factors such as previous pregnancy or operative approach. Although the power of our study was 70% due to the rarity of CP, our study contributes valuable knowledge to the existing literature, particularly regarding the patient baseline characteristics within the Thai population.
